# Intrahepatic cholangiocarcinoma hidden within cancer of unknown primary

**DOI:** 10.1038/s41416-022-01824-4

**Published:** 2022-04-28

**Authors:** Alicia-Marie Conway, Georgina C. Morris, Sarah Smith, Monique Vekeria, Prakash Manoharan, Claire Mitchell, Alison Backen, Pedro Oliveira, Richard A. Hubner, Angela Lamarca, Mairéad G. McNamara, Juan W. Valle, Natalie Cook

**Affiliations:** 1 Cancer Research UK Manchester Institute Cancer Biomarker Centre, The University of Manchester, Manchester, UK; 2 Department of Medical Oncology, The Christie NHS Foundation Trust, Manchester, UK; 3 School of Medical Sciences, University of Manchester, Manchester, UK; 4 Department of Radiology, The Christie NHS Foundation Trust, Manchester, UK; 5 Division of Cancer Sciences, University of Manchester, Manchester, UK; 6 Department of Pathology, The Christie NHS Foundation Trust, Manchester, UK

**Keywords:** Cancer models, Cancer genomics

## Abstract

**Background:**

Many patients referred with a provisional diagnosis of cancer of unknown primary (pCUP) present with presumed metastatic disease to the liver. Due to the lack of definitive histological markers, intrahepatic cholangiocarcinoma (iCCA) may be overlooked. This study assessed the frequency of iCCA within a pCUP cohort.

**Methods:**

A single UK cancer-center study of sequential patients referred with pCUP from January 2017 to April 2020. Baseline diagnostic imaging was reviewed independently by a radiologist and oncologist; those with radiological features of iCCA (dominant liver lesion, capsular retraction) were identified.

**Results:**

Of 228 patients referred with pCUP, 72 (32%) had malignancy involving the liver. 24/72 patients had radiological features consistent with iCCA; they were predominantly female (75%) with an average age of 63 years and 63% had an ECOG PS ≤ 2. The median overall survival (OS) of the iCCA group and the remaining liver-involved CUP group were similar (OS 4.1 vs 4.4 months, *p*-value = 0.805). Patients, where a primary diagnosis was subsequently determined, had better OS (10.2 months, *p*-values: iCCA = 0.0279: cCUP = 0.0230).

**Conclusions:**

In this study, 34% of patients with liver-involved pCUP, fulfilled the radiological criteria for an iCCA diagnosis. Consideration of an iCCA diagnosis in patients with CUP could improve timely diagnosis, molecular characterisation and treatment.

## Background

Cancer of Unknown Primary (CUP) is a collective term encapsulating metastatic cancers for which the primary site of origin remains elusive, despite thorough clinical, radiological and histopathological review. Although the incidence of CUP is declining, potentially reflecting improvements in diagnostics leading to the determination of the primary tumour site, it remains the 6th leading cause of cancer death in the UK with a median survival of 6–16 months [1, 2]. In the UK, patients presenting with metastatic disease where the primary site is not immediately apparent have a provisional diagnosis of pCUP [3] and should be treated on a CUP pathway, which includes discussion at a CUP specialist multi-disciplinary team meeting (MDT). Following appropriate radiological and pathological review patients with no clear primary are given a diagnosis of confirmed CUP (cCUP). Of those with cCUP, around 20% of patients have clinicopathological features that resemble that of a known tumour type and fall within a “favourable” subset. These patients, in line with European Society of Medical Oncology (ESMO) CUP guidelines [4], should be treated according to the linked tumour type, with access to molecular subtyping and treatments including immunotherapy and targeted agents and have survival similar to the well-known tumour type in the metastatic setting [5]. For example, women with isolated axillary node metastasis from adenocarcinoma should be treated on a breast cancer pathway and patients with adenocarcinoma with a lower gastrointestinal profile should be treated as colorectal cancer [4]. Unfortunately, the remaining 80% of patients with CUP make up an “unfavourable” subset with a much poorer prognosis. Treatment options are limited to first-line doublet chemotherapy and there are no standard second-line treatments. This ‘one-size-fits-all’ approach does not reflect the clinical, pathological and molecular heterogeneity of these tumours and emphasises the need for better patient stratification in the unfavourable subset.

Despite major advances in molecular subtyping and treatments in a number of malignancies, there has been little advance in CUP, despite epitomising the need for a precision medicine approach. Molecular profiling of CUP has revealed diverse genetic heterogeneity and potentially therapeutically targetable mutations [6–8]. However, without primary tumour determination, the actionability of mutations and access to profiling and treatments is very limited [9].

Determining the tissue of origin via molecular profiling in patients with CUP could potentially identify patients with chemotherapy-responsive tumour types, who may have better outcomes with more tailored therapies [10, 11]. However, recent randomised controlled trials have failed to show improved clinical outcomes by treating patients based on molecular tissue of origin predictions [12, 13]. Trials are, however, hampered by the heterogeneity of the disease, access to targeted therapies, lack of statistical power for subtype analysis and long recruitment, which often means disease-specific treatment regimens are outdated. It is noteworthy that often the subgroup analysis is too small to draw conclusions, but collectively there is evidence of more favourable subsets emerging [14]. For example, the colorectal cancer-CUP (CRC-CUP) entity emerged through molecular profiling trials and these patients now survive comparably to patients with metastatic colorectal cancer on tailored chemotherapy [15, 16]. New favourable subsets are emerging, often in response to improved chemotherapy regimens in cancer types, they align to and/or the emergence of efficacious targeted therapies [14]. With targeted and immuno- therapies superseding chemotherapy in many metastatic tumour settings, and significantly improving survival, it is imperative to identify patients with CUP with treatable tumours and it is partly through access to molecular profiling that these subsets are becoming more easily recognised. For patients with CUP and targetable alterations where new therapeutic options are emerging, identification of tissue of origin is even more important.

One such emerging favourable CUP subset is intrahepatic cholangiocarcinoma (iCCA); a rare cancer entity arising from the biliary epithelium likely to be overrepresented in CUP cohorts due to the challenges associated with its diagnosis [17]. The diagnosis of iCCA is difficult to differentiate pathologically from other tumour types, such as pancreatic and upper gastrointestinal carcinomas, due to the lack of specific immunohistochemical biomarkers. However, there are distinct radiological appearances that can be used for iCCA diagnosis [18]. In this study, we sought to identify patients with clinicopathological features in keeping with iCCA from a cohort of patients provisionally diagnosed with CUP (pCUP).

## Methods

### Patient population and data collection

Permission was granted to collect retrospective patient data for this study by the Quality Improvement and Clinical Audit Committee at a single institution (The Christie NHS Foundation Trust; reference number 2515; 17 April 2019). Ethical approval for subsequent tissue analysis was obtained from Yorkshire & The Humber—Sheffield Research Ethics Committee (REC; reference approval ID 20/YH/0305). Data were collected from a sequential cohort of patients referred to the centralised CUP service with a provisional diagnosis of CUP between the period of 01.01.2017–01.04.2020.

Data were collected from institutional electronic patient records and standardised according to criteria described in Supplementary Fig. 1. Relevant patient demographics were captured including age, gender, metastatic burden with respect to liver involvement, Eastern Oncology Cooperative Group (ECOG) performance status (PS), treatment, date of diagnosis, and date of death or last recorded visit. The number and types of multi-disciplinary team (MDT) meetings where patients were discussed were also captured. Histology reports were reviewed and, where documented, the results from immunohistochemistry staining were collected. Patients were classified based on the site of documented disease as “No Liver Involvement” or “Liver Involvement”. After a retrospective radiological review of the images of patients with liver involvement, those with a potential iCCA diagnosis were determined and are defined as our iCCA cohort. The final diagnosis of all remaining patients referred with pCUP was recorded as the subsequent outcome at the end of CUP pathway and MDT decision(s): either as a primary tumour diagnosis or confirmed CUP (cCUP). Patients with a high suspicion of a primary tumour diagnosis within a favourable CUP subgroup as classified by ESMO CUP guidelines were categorised as a primary tumour diagnosis for the purpose of this study.

### Retrospective radiological evaluation

To identify the iCCA cohort, all patients with liver involvement had two independent blinded retrospective radiological assessments performed by a specialist hepatobiliary medical oncologist and specialist gastrointestinal (GI) radiologist, respectively. Both radiologists and oncologists were blinded to the clinical information of patients, final tumour diagnosis, histology and reciprocal conclusions of the radiology review. Detailed radiological features were recorded for each patient including imaging modality, liver lesion characteristics, associated radiological features and extrahepatic metastatic sites of disease. The radiological features used to determine iCCA classification are outlined in Supplementary Fig. 1. Radiological examples of iCCA and non-iCCA are shown in Supplementary Fig. 2.

A diagnosis of iCCA was made when both radiologist and oncologist agreed there was radiological evidence of iCCA or “possible” iCCA. Patients with iCCA and “possible” iCCA were pooled for analysis as the “iCCA cohort”, with the remainder of patients forming the confirmed CUP (cCUP) liver-involved or liver-involved primary diagnosis subgroups. Where radiology was non-evaluable, these patients were presumed to have a cCUP diagnosis, unless a clear primary tumour was documented in the case notes.

### Molecular profiling

The time frame for patients reviewed in this study pre-dates the introduction of the standard of care molecular profiling for patients with iCCA; however, we sought to perform this retrospectively to establish the frequency of potentially targetable alterations in the iCCA cohort. Two patients had molecular profiling performed as part of a trial enrolment [19, 20]. For those patients without molecular profiling performed, the remaining diagnostic tissue was retrospectively evaluated for tumour content by a CUP pathologist. Those samples with adequate remaining tissue underwent DNA extraction and genomic profiling in a Clinical Laboratory Improvement Amendments-certified, College of American Pathologists-accredited, New York State-approved laboratory (Foundation Medicine, Cambridge, MA), using FoundationOne CDx tissue assay (Roche, Foundation Medicine) [21].

### Data analysis and statistical tests

Subgroup analysis was performed on all data from patients with liver involvement; iCCA, cCUP and primary tumour diagnosis. Survival analysis was performed using GraphPad Prism version 9.0.0 for Windows, GraphPad Software, San Diego, California USA. Kaplan–Meier Curves and Log-Rank (Mantel-Cox) tests were performed, with adjusted *p*-value significance to Bonferroni corrected threshold, where appropriate. All patients were followed up until death or the time of data lock on 7 April 2021. Overall survival was calculated from the date of diagnosis to the date of death or censored at the date of the last contact.

## Results

Two-hundred and twenty-eight patients were included for review with a provisional CUP (pCUP) diagnosis. One patient was excluded as their final diagnosis was non-cancerous (chronic osteomyelitis). The average age of the cohort was 68 years (range 26–93), 112 (49%) were female and 115 (50%) had an ECOG PS of 0-1. Fifty-two (22%) patients were diagnosed with a non-iCCA primary tumour diagnosis as a final diagnosis.

**Fig. 1:**
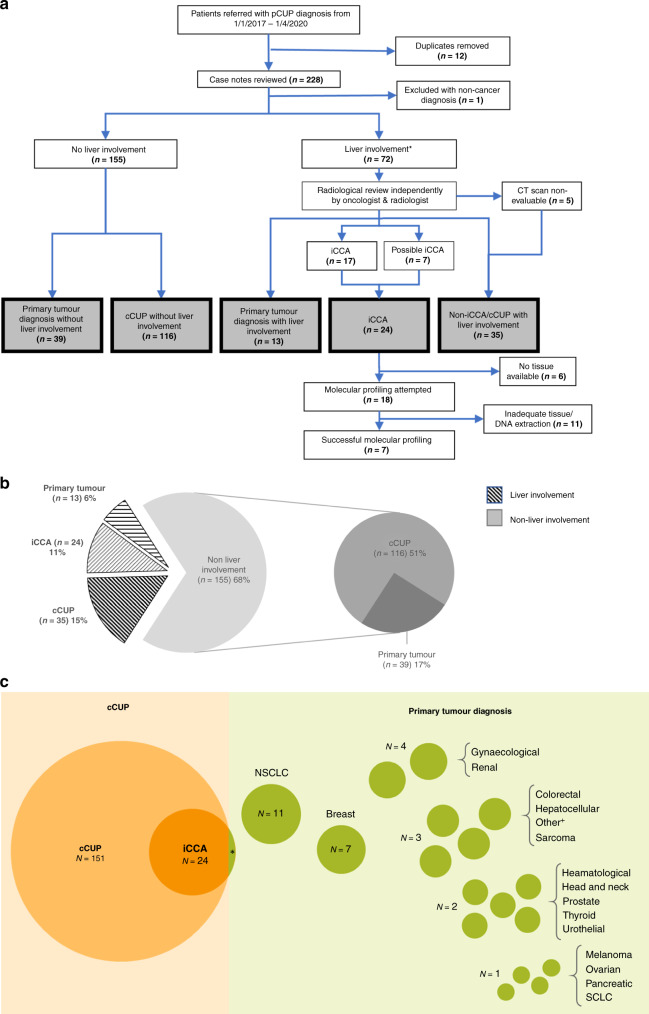
Consort diagram, cohort summary and summary of patient final diagnoses. **a** Consort diagram of cohort with final diagnosis highlighted in grey. **b** Split of whole cohort of patients by final diagnosis. **c** Proportional representation of final diagnosis of all cancer patients referred with provisional CUP. Circle areas are scaled to represent number of patients. *N = 1 patient had a real-time primary diagnosis of iCCA. +Other cancers were Myxopapillary ependymoma, metastatic extra-mammary Paget’s disease and Langerhans cell histiocytosis. *8 patients had liver as only site of disease. cCUP confirmed Cancer of Unknown Primary, iCCA intrahepatic cholangiocarcinoma, cCUP confirmed Cancer of Unknown Primary, pCUP provisional Cancer of Unknown Primary, iCCA intrahepatic cholangiocarcinoma, CT computer tomography, NSCLC non-small cell lung cancer, SCLC small cell lung cancer.

Liver involvement was present in 72 (32%) patients; there was no liver involvement in the remaining 155 (68%) patients. Of those with no liver involvement, 116 were found to have confirmed CUP (cCUP no liver involvement) and in 39 patients, a primary tumour diagnosis was made (Fig. 1a). All patients with liver involvement (*n* = 72) went on to have radiological reviews. Of these patients, 24 were identified to have an iCCA diagnosis (in all, except one patient, the diagnosis was made on retrospective radiological review). Of the remaining 48 patients, 13 had a non-iCCA primary tumour diagnosis and 35 had confirmed CUP (cCUP liver involvement) (Fig. 1b). Twenty different primary tumour types were diagnosed during the CUP pathway in 53 patients (23%). The most common diagnoses made were breast cancer, gynaecological malignancy, renal cell carcinoma and non-small cell lung cancer (NSCLC) (Fig. 1c).

### Demographics

Patients with iCCA were on average younger and had a poorer ECOG PS, when compared with those with cCUP and liver involvement, as summarised in Table 1. They were also statistically more likely to be female; 75% compared to 43% in cCUP liver involvement group; *p*-value = 0.0181, two-sided Fisher exact test. More than 90% of all patients were discussed in at least one MDT, predominantly a CUP-dedicated MDT (range from 56 to 92%). Of note across the cohorts, the proportion of patients where cases had been discussed at 2 or more different MDT meetings was between 50% (iCCA cohort) and 62% (primary tumour diagnosis without liver involvement). Nine patients (23%) within the iCCA cohort were discussed at a hepatobiliary MDT during the CUP pathway, only one of these patients had an iCCA diagnosed at this time (Table 1).

**Table 1:** Cohort characteristics.

		Liver involvement			No liver involvement	
		cCUP	iCCA	Primary tumour diagnosis	cCUP	Primary tumour diagnosis
		(*N* = 35)	(*N* = 24)	(*N* = 13)	(*N* = 116)	(*N* = 39)
Gender	Female	15 (43%)	18 (75%)	4 (31%)	47 (41%)	28 (72%)
	Male	20 (57%)	6 (25%)	9 (69%)	69 (59%)	11 (28%)
ECOG performance status	0	2 (6%)	3 (13%)	2 (15%)	13 (11%)	7 (18%)
	1	13 (37%)	6 (25%)	6 (46%)	46 (40%)	16 (41%)
	2	12 (34%)	6 (25%)	4 (31%)	20 (17%)	11 (28%)
	3	6 (17%)	8 (33%)	1 (8%)	28 (24%)	5 (13%)
	4	2 (6%)	1 (4%)	0	9 (8%)	0
Age	Mean	69	63	68	69	70
	Range	36–84	31–79	29–82	26–93	44–87
MDT discussions	Any MDT	35 (100%)	24 (100%)	12 (92%)	111 (96%)	36 (92%)
	≥2 MDTs	19 (54%)	12 (50%)	7 (54%)	71 (61%)	24 (62%)
	CUP MDT	25 (71%)	22 (92%)	9 (69%)	82 (71%)	22 (56%)
	HPB MDT	10 (29%)	9 (36%)	3 (23%)	5 (4%)	0

*cCUP* confirmed Cancer of Unknown Primary, *iCCA* intrahepatic cholangiocarcinoma, *ECOG* Eastern Cooperative Oncology Group, *MDT* multi-disciplinary team meeting, *HPB* hepatobiliary.

### Histology

**Fig. 2:**
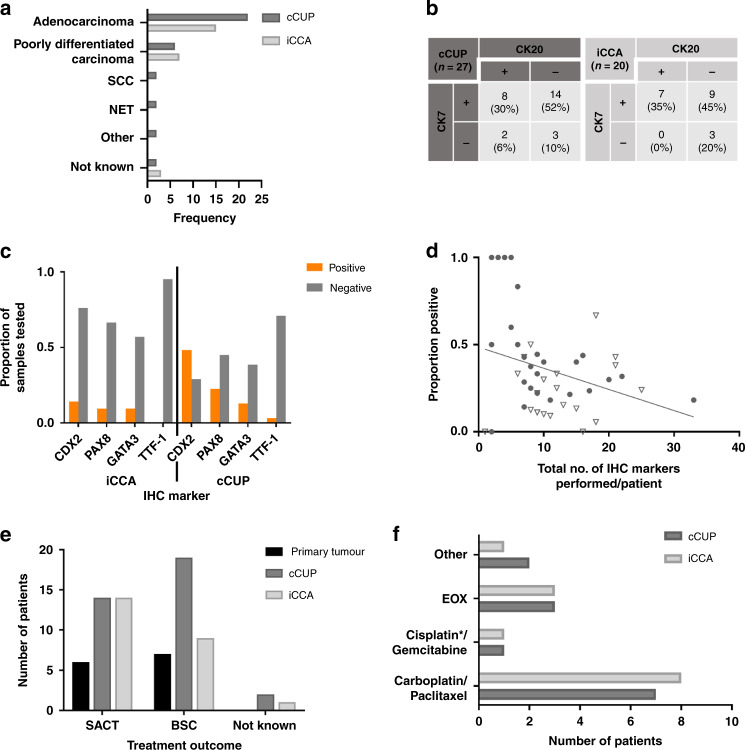
Characteristics and treatment outcomes of patients with liver involvement and confirmed CUP (cCUP), intrahepatic cholangiocarcinoma (iCCA) or primary tumour final diagnosis. **a** Histological frequency of patients with liver involvement and final diagnosis of cCUP (*N* = 36) or iCCA (*N* = 25). **b** Cytokeratin 7 (CK7) and Cytokeratin 20 (CK20) staining patterns for those patients with liver involvement and a final diagnosis of cCUP and iCCA, where an immunohistochemistry (IHC) result was documented. **c** Proportion of documented IHC positivity for IHC markers caudal-type homeobox 2 (CDX2), paired box gene 8 (PAX8), GATA binding protein 3 (GATA3) and transcription termination factor 1 (TTF-1) for patients with liver involvement and cCUP and iCCA. **d** Combined results for cCUP (circle) and iCCA (triangle) patients with liver involvement showing the total number of IHC tests performed per patient versus the proportion of these that were positive. **e** Treatment outcomes for patients with liver involvement (*N* = 74). SACT systemic anti-cancer therapy, BSC Best Supportive Care. **f** Systemic anti-cancer therapy regimens received by patients with cCUP and iCCA. cCUP confirmed CUP, iCCA intrahepatic cholangiocarcinoma. *1 patient received carboplatin with gemcitabine.

All patients whose tumours were determined as iCCA, with documented histology, had a histological profile compatible with iCCA: either adenocarcinoma (*n* = 14) or poorly differentiated carcinoma (*n* = 7). Most patients with liver-involved cCUP were also adenocarcinomas or poorly differentiated carcinomas; however, a few patients had other histological subtypes (Fig. 2a).

Documented results were available for the immunohistochemical (IHC) markers cytokeratin 7 (CK7) and cytokeratin 20 (CK20) in 27 patients (77%) with cCUP liver involvement and 19 patients (79%) within the iCCA group. All patients with iCCA showed CK7 positivity. Generally, tumours were CK20 negative, but not consistently, and a similar proportion of patients with cCUP showed a CK7 positive/CK20 negative IHC pattern (Fig. 2b). However, patients with iCCA demonstrated more negativity for markers such as CDX2, PAX8, GATA3 and TTF-1, in contrast to cCUP (Fig. 2c). For patients with liver involvement with a final diagnosis of cCUP or iCCA, the median number of IHC markers performed was 9 (range: 0–33). Twenty four patients (39%) had more than 10 IHC markers performed. As expected, the number of IHC markers evaluated against the proportion of positive IHC results shows a negative correlation (Spearmans R^2^ = −0.3118; 95% Confidence Interval (CI) −0.5535 to −0.02175; two-tailed *p*-value = 0.0310) (Fig. 2d), highlighting the limited value of numerous IHC attempts in these tumours.

### Radiology review

Radiology review was performed in 67/72 patients with liver involvement, with five patients scans non-evaluable. The majority of patients (96%) had a contrast CT as a radiological modality, with seven patients having an additional liver MRI, two patients only had MRI of liver and one patient had only at PET CT scan performed. The radiological features of the 24 patients determined to be iCCA are summarised in Supplementary Table 1. All but two patients demonstrated a single dominant liver lesion with at least 5 cm largest axial dimension. All patients had a heterogenous mass with irregular margins and central hyperintensity with peritumoural enhancement. The majority of patients (22/24) had satellite liver nodules in addition to a dominant liver lesion and half of the patients demonstrated vascular encasement. Liver capsular retraction was present in 10 patients and dilated intrahepatic bile ducts in 6 patients. Liver cirrhosis, tumour thrombus and portal hypertension were not commonly associated radiological features. Almost all patients had extrahepatic metastatic sites of disease, with just under half (*n* = 11) having visceral metastasis present. Of the 72 patients with liver involvement eight patients were identified with liver-only visceral disease; 7/8 had portal node involvement and only one had disease completely confined to the liver. Of these eight patients with the liver-only disease, seven were retrospectively determined as having iCCA; the remaining patient had a primary hepatocellular tumour diagnosed during the CUP pathway work-up. Radiologist and oncologist review for determining iCCA was generally concordant. In only one case were the radiologist and oncologist entirely discordant. For four patients “possible iCCA” was considered by one reviewer but deemed not to be iCCA by the other; these patients were not included within the iCCA cohort.

### Treatment decisions

For patients with documented treatment outcomes, patients with iCCA were more often managed with systemic anti-cancer therapies (SACT) than best supportive care (BSC) when compared to patients with cCUP liver involvement (58% vs 40%, respectively) (Fig. 2e). There was no statistically significant difference in overall survival (OS) between the patients with iCCA and cCUP with liver involvement that received SACT (Supplementary Fig. 3A). For both cohorts, the most common chemotherapy regimen given was carboplatin/paclitaxel (Fig. 2f). Only one of the patients with iCCA received the current gold-standard first-line chemotherapy combination for iCCA, cisplatin/gemcitabine. Those with a final diagnosis of cCUP who had carboplatin-based chemotherapy (*n* = 8) had a numerically longer median OS when compared to the iCCA group receiving the same chemotherapy (*n* = 7) (8.9 vs 5.1 months, log-rank (mantel-Cox) *p*-value = 0.7387) (Supplementary Fig. 3B); however, this did not reach statistical significance. For the iCCA cohort, 3 patients went on to have second-line therapy.

### Mutational analysis

**Fig. 3:**
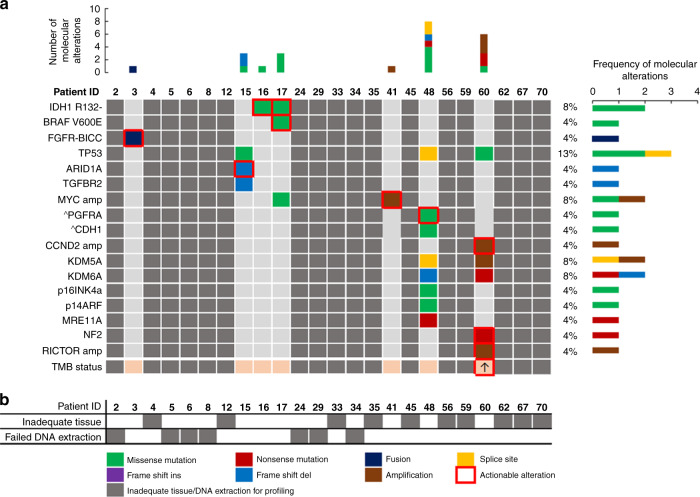
Mutation profiling of intrahepatic cholangiocarcinoma (iCCA) cohort. **a** Oncoplot of number, type and frequency of mutations by patient as reported by Foundation Medicine, actionable mutations highlighted in red box. Greyed out if mutation profiling unavailable; ^subclonal mutation. *patient underwent local molecular profiling rather than Foundation Medicine assay. **b** Reasons for mutation profiling failure by patient. White = successful profiling, Grey = unsuccessful. ARID1A AT-rich interaction domain 1 A, BICC bicaudal C Homolog 1, BRAF proto-oncogene B-raf, CCND2 cyclin D2, CDH1 cadherin 1, FGFR fibroblast growth factor receptor, IDH isocitrate dehydrogenase, KDM5A lysine demethylase 5 A, KDM6A lysine demethylase 6 A, MRE11A MRE11 Homolog, Double Strand Break Repair Nuclease, MYC MYC proto-oncogene, NF2 neurofibromin 2, P14ARF cyclin dependent kinase inhibitor 2 A, P16INK4a cyclin dependent kinase inhibitor 2 A, PGFRA platelet-derived growth factor receptor alpha, RICTOR RPTOR independent companion of MTOR Complex 2, TGFBR2 transforming growth factor beta receptor 2; TMB tumour mutation burden.

As molecular profiling was not offered routinely in patients with cCUP or iCCA during the study period, only two patients within iCCA cohort had access to this. Both patients had molecular alterations consistent with iCCA diagnosis (One isocitrate dehydrogenase (IDH)1 mutation; one fibroblast growth factor receptor (FGFR)2 fusion). Of the remaining 22 patients with iCCA, 16 had enough residual diagnostic tissue to attempt retrospective molecular profiling (Fig. 1a). Four of these samples failed external pathology review and had inadequate tissue for molecular profiling and a further seven samples failed profiling due to inadequate quality or quantity of DNA extracted. Of the five remaining patients that were successfully profiled retrospectively all had mutations detected that could be considered consistent with an iCCA diagnosis (Fig. 3) and all potentially actionable with licensed therapies or those in late-phase trials.

### Survival statistics

**Fig. 4:**
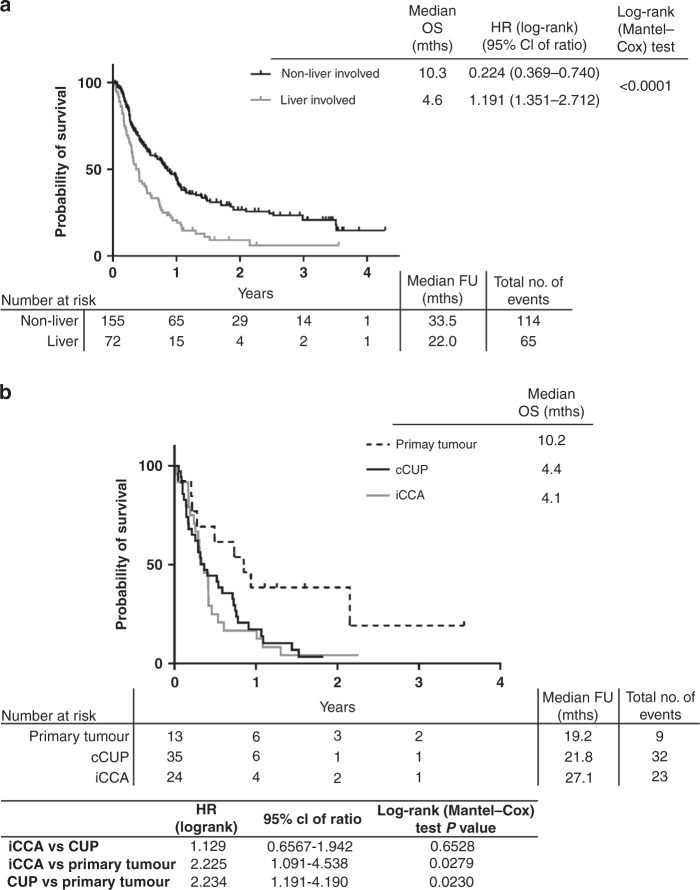
Survival curves of cohort. **a** Kaplan–Meier curves for overall survival (OS) of whole cohort (excluding 1 patient with non-cancer diagnosis) split by liver involvement. **b** Kaplan–Meier curve for OS of those patients with liver involvement only, split by final diagnosis. CUP Cancer of Unknown Primary, iCCA intrahepatic cholangiocarcinoma, FU follow-up, mths months.

Overall survival of the whole cohort of patients demonstrated those with liver involvement (*n* = 72) had significantly poorer OS than those without liver involvement (*n* = 155) (4.6 months vs. 10.3 months, log-rank (Mantel-Cox) test *p*-value < 0.0001, Fig. 4a). In the cohort of patients with liver involvement, the median OS was longer in patients with a non-iCCA primary tumour diagnosis (*n* = 13; median OS = 10.2 months), compared with patients with iCCA (*n* = 24; median OS = 4.1 months; log-rank (Mantel-Cox) test *p*-value 0.0279) or liver-involved cCUP (4.4 months; log-rank (Mantel-Cox) test *p*-value 0.0230) as the final diagnosis (Fig. 4b), although this was not statistically significant with a Bonferroni adjusted *p*-value threshold of 0.017.

## Discussion

A previously poor prognostic cancer type, iCCA is now known to show distinctive molecular aberrations and targetable molecular changes; most notable are translocations of fibroblast growth factor receptor 2 (FGFR2) and, isocitrate dehydrogenase (IDH) 1/2 mutations. IDH1/2 mutations have been reported in 10–36% of iCCA tumours and FGFR2 fusions in 11–45% [22–28]. There is now encouraging clinical trial evidence of the efficacy of multiple targeted therapies in iCCA. Ivosidenib shows efficacity in IDH1 mutant cholangiocarcinoma [29] and BRAF inhibitors dabrafenib and trametinib in patients with BRAF V600E mutations [30]. FGFR inhibitors pemigatinib and infigratinib, are both effective in patients with FGFR2 fusions with pemigatinib recently approved in the second-line setting for patients with iCCA [31, 32]. This highlights the increased need to identify patients with CUP that should be re-classified as iCCA so appropriate molecular testing can be performed for appropriate treatment stratification.

To our knowledge, this dataset is the first to seek to identify the frequency of iCCA within a large pCUP cohort retrospectively. Although iCCA is recognised as being overrepresented in CUP cohorts [17], it remains a rare entity and a diagnosis of exclusion usually requiring specialist input, and therefore may be overlooked as a potential diagnosis. Due to a lack of specific histological biomarkers to indicate an iCCA diagnosis, recognition of characteristic radiological imaging of iCCA, along with supportive histopathology appearances, are currently the only way to make this diagnosis.

Within this current dataset, 24 patients were identified retrospectively with radiological evidence of iCCA. This equated to 11% of all patients referred with pCUP, 16% of patients where their end referral pathway diagnosis was cCUP and 41% of cCUP patients with liver involvement. Only one of these patients was identified as iCCA during the CUP pathway but all had histological and radiological compatibility with an iCCA diagnosis. This emphasises the need for further subtyping of patients with CUP with liver involvement by radiological features and consideration of iCCA as a potential diagnosis in these patients.

The proportion of patients with iCCA identified in this cohort is comparable to gene expression profiling studies that suggest up to 21% of patients with CUP patients have a gene expression profile compatible with a biliary tract cancer [10, 13]. In a separate, but large case series of patients with CUP, a similar proportion of iCCA was confirmed (22%) using albumin RNA in situ hybridisation [33]. We can compare this to recent preliminary results from the ongoing CUPISCO trial (NCT03498521); a phase II study comparing the molecularly guided therapy compared to standard chemotherapy in good performance status patients with “unfavourable” CUP. It demonstrated out of 628 patients screened for recruitment as of April 2020, 5.7% of patients had radiological and pathological features of iCCA after an extensive review of radiology and pathology [20]. This lower proportion compared to other studies, and our own, likely reflects the inclusion criteria for recruitment of an ECOG PS of 0-1 and prior specialist review of radiology and pathology [20]. Of note, several large tissues of origin studies in CUP do not have iCCA as a potential tumour type, and in some cases it is grouped with the pancreato-biliary cancer type, demonstrating a limitation with some existing tissue of origin classifiers and the need for rare tumours and tumour subtypes to be included in such classifiers [13, 34, 35].

In this current dataset, the iCCA group were predominantly female and of slightly younger age. Only one patient in the iCCA group received the standard of care first-line cisplatin/gemcitabine chemotherapy [36], with the majority receiving carboplatin/paclitaxel. This may reflect the female predominance of this cohort and the potential inclination of clinicians to give a regimen commonly used in female gynaecological cancers. Although the patient numbers are small, comparing the survival of 15 patients that had carboplatin/paclitaxel (8 cCUP and 7 iCCA), there was no significant difference in OS, however, there was a trend of poorer survival in the iCCA group (Supplementary Fig. 3b).

Amongst patients with liver involvement and a diagnosis of either cCUP or iCCA, often multiple IHC markers (10+) had been performed but were rarely positive or identified as a primary tumour. Performing multiple IHC tests is time-consuming, can contribute to delays in treatment/eligibility for therapy and exhausts valuable material that may be better reserved for molecular profiling, especially in light of emerging targeted treatments in iCCA. In the small proportion of patients with tissue available for profiling (seven) we confirmed four patients within our iCCA cohort had molecular alterations (IDH1 R132* mutations, BRAF V600E and an FGFR translocation) that are now targetable with drugs approved in iCCA or showing efficacy in late-phase trials [29–31, 37, 38].

Patients with cCUP may benefit from molecular profiling, with recent reports demonstrating up to 90% of CUP tumours harbour potentially targetable mutations [6, 7, 39, 40]. However, only small numbers of patients with CUP have been treated with targeted therapies based on molecular profiling [41]. Given molecular profiling is now available as a standard of care for many primary tumours, and 22% of patients referred with pCUP ended up with a primary tumour diagnosis, it would be reasonable to propose future guidelines should consider upfront molecular profiling for all patients with pCUP. This may enable more timely diagnosis and treatment decisions and prevent exhaustion of valuable tissue. For patients with available tissue, biopsy material is invariably scarce and repeat biopsies are often difficult to obtain. This is exemplified by the CUPISCO trial data suggesting that up to 25% of recruited patients screen failed due to a lack of tissue quantity/quality for diagnosis confirmation and sequencing [20]. As we saw from our iCCA cohort only 7/24 (29%) patients had sufficient material for successful molecular profiling, which may be a result of these samples being from archival diagnostic biopsy specimens. Blood-based diagnostics utilising circulating tumour DNA (ctDNA) for molecular profiling can overcome the limitations of the need for tissue biopsies and is feasible in patients with CUP [19, 42], providing a potential alternative for diagnosis/profiling, where tissue is scarce.

Over half (56%) of the whole cohort had been discussed in two or more MDT meetings, with the highest proportion occurring in the patients with a primary tumour diagnosis without liver involvement, perhaps a reflection of the uncertain presentation of these primary tumours and hence referral as a pCUP. For all patients multiple MDT discussions inevitably lead to delays in final diagnosis and treatment initiation. Overall, this highlights the difficulty in diagnosing these patients with radiology alone, even within specialist MDTs, and emphasises an ongoing clinical need for better diagnostics and biomarkers to confirm or exclude an iCCA diagnosis. In patients with liver-dominant CUP and radiological appearances most clinically suspicious for cholangiocarcinoma, the treating clinicians should manage the patient as an iCCA.

**Fig. 5:**
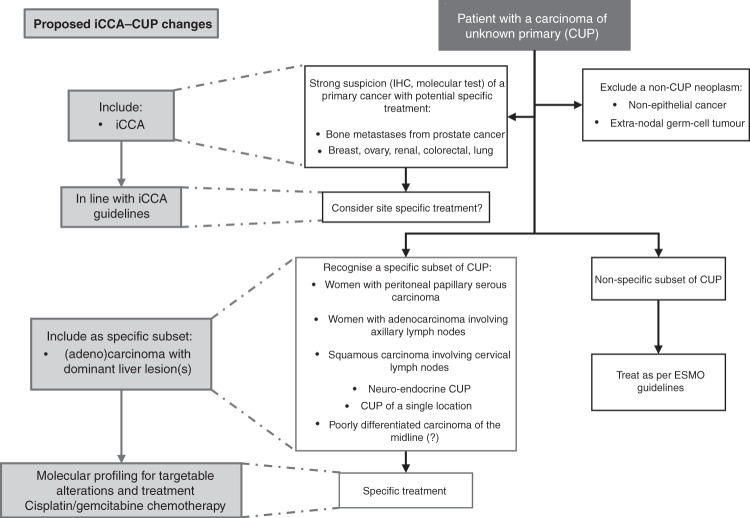
Proposed inclusion of intrahepatic cholangiocarcinoma-Cancer of Unknown Primary (iCCA-CUP) favourable subset within current European Society of Medical Oncology (ESMO) guidelines (3). Inclusion of ‘(adeno)carcinoma with dominant liver lesion(s)’ as specific favourable subset for patients to be treated in line with iCCA guidelines with access to molecular profiling and disease-specific treatments. IHC immunohistochemistry.

Careful radiological and pathology evaluation, comprehensive clinical information and access to molecular profiling may enable a confident diagnosis of iCCA in patients with liver-involved CUP and could enable some of these patients to receive targeted therapies, now licensed in iCCA. To overcome the current diagnostic challenges of identifying iCCA within CUP cohorts, as described here, we propose the introduction of a ‘liver-dominant’ CUP subset into future CUP guidelines, to enable timely identification of potential iCCA diagnoses. All patients with a CT scan demonstrating liver-only or dominant liver lesions with a compatible radiological and histological profile should be considered as iCCA if no alternative diagnosis is apparent. These patients should have access to molecular profiling to identify any actionable targets and should be treated in line with iCCA clinical guidelines (Fig. 5).

## Supplementary information

Supplementary figures

AJ Checklist

## Data Availability

All anonymised patient data will be available from the corresponding author.
