# Supplementary material for: Intrahepatic cholangiocarcinoma hidden within cancer of unknown primary
Source: Br J Cancer. 2022 Apr 28;127(3):531–40. doi: 10.1038/s41416-022-01824-4 (PMC9345855; doi:10.1038/s41416-022-01824-4)
Supplement: Supplementary file 1 — Supplementary figures [file 41416_2022_1824_MOESM1_ESM.pdf]

## Supplementary Material

**Supplementary Table 1. Intrahepatic cholangiocarcinoma cohort radiological characteristics and summary.** iCCA=intrahepatic cholangiocarcinoma; CT=computerised tomography; MRI=magnetic resonant imaging; Art=arterial phase; PV=portal venous phase

**Supplementary Figure 1**. A) Guidelines for clarification & standardization of data. B) Radiological definition for retrospective classification of iCCA. iCCA – intrahepatic cholangiocarcinoma; CT – computed tomography *Includes iCCA/HCC Combined Tumours - a rare but distinct tumour based mainly on histology findings.

**Supplementary Figure 2**. A) Radiological example of intrahepatic cholangiocarcinoma (iCCA) demonstrating a dominant hypodense liver lesion and capsular retraction (arrows). B) Radiological example of bi-lobar liver metastases from a solid tumour primary.

**Supplementary Figure 3:** A) Kaplan-Meier curves for overall survival (OS) of all patients with liver-involvement who undertook systemic anti-cancer treatment (SACT). No significant difference in survival between groups. B) Kaplan-Meier curve for survival for the 15 patients who received carboplatin/paclitaxel chemotherapy, split by confirmed cancer of unknown primary (cCUP) or intrahepatic cholangiocarcinoma (iCCA) diagnosis. No significant difference in OS (Log-Rank (Mantel-Cox) test p-value=0.7387).

**Supplementary Table 1:** Intrahepatic cholangiocarcinoma cohort radiological review summary

| | Radiology review | | Radiology modality | | Hepatic tumour features | | | | | Associated radiological features | | | | | | | |
|---|---|---|---|---|---|---|---|---|---|---|---|---|---|---|---|---|---|
| Case ID | Oncologist review | Radiologists review | Radiology modality | Phase of CT | Single dominant liver lesion? | Largest axial dimension (cm) | Mass description | Central hypointensity | Peritumoural enhancement | Capsule Retraction | Dilated Intrahepatic Bile Ducts | Satellite liver nodules | Vascular encasement | Tumour Thrombus | Cirrhosis | Portal Hypertension | Extrahepatic metastatic sites |
| 2 | Possible iCCA | iCCA | CT | PV only | Present | 5.1 | Heterogenousmass with irregular margin | Present | Present | Absent | Absent | Present | Absent | Absent | Absent | Absent | Portal nodal mass |
| 3 | iCCA | iCCA | CT | Art, PV | Present | 7 | Heterogenous mass with irregular margin | Present | Present | Absent | Absent | Present | Present | Present | Absent | Absent | Portal, lung |
| 4 | Possible iCCA | Possible iCCA | CT | Art, PV | Present | 14 | Heterogenous mass with irregular margin | Present | Present | Absent | Absent | Present | Absent | Absent | Present | Absent | Bone, adrenal |
| 5 | iCCA | iCCA | CT and MRI | PV only | Absent | n/a | Heterogenous mass with irregular margin | Present | Present | Absent | Absent | Present | Present | Absent | Absent | Absent | None |
| 6 | iCCA | iCCA | CT | PV only | Present | 12 | Heterogenous mass with irregular margin | Present | Present | Present | Absent | Present | Absent | Absent | Absent | Absent | Nodes |
| 8 | iCCA | iCCA | CT | Art, PV | Present | 15 | Heterogenous mass with irregular margin | Present | Present | Present | Absent | Present | Present | Absent | Absent | Absent | Portal nodes |
| 12 | iCCA | Possible iCCA | CT | PV only | Present | 14 | Heterogenous mass with irregular margin | Present | Present | Absent | Absent | Present | Absent | Absent | Absent | Absent | Portal and mesenteric nodes |
| 15 | Possible iCCA | iCCA | CT | triple phase | Present | 13 | Heterogenous mass with irregular margin | Present | Present | Present | Absent | Present | Present | Absent | Absent | Absent | Portacacal nodes and perihepatic ascities |
| 16 | iCCA | iCCA | CT | PV only | Present | 8 | Heterogenous mass with irregular margin | Present | Present | Present | Absent | Present | Present | Absent | Absent | Absent | Bone, nodal, peritoneal nodules and small pelvic ascities |
| 17 | iCCA | iCCA | CT | Art, PV | Present | 6 | Heterogenous mass with irregular margin | Present | Present | Absent | Absent | Present | Present | Absent | Absent | Absent | Bone, nodal, lung, peritoneal nodules and small pelvic ascities |
| 24 | Possible iCCA | Possible iCCA | CT | Art, PV | Present | 5.6 | Heterogenous mass with irregular margin | Present | Present | Absent | Present | Present | Present | Absent | Absent | Absent | Hilar nodes, small volume ascities |
| 29 | iCCA | iCCA | CT | Art, PV | Present | 13 | Heterogenous mass with irregular margin | Present | Present | Absent | Absent | Present | Absent | Absent | Absent | Absent | Nodes and perihepatic ascities |
| 33 | iCCA | iCCA | CT | PV only | Present | 13 | Heterogenous mass with irregular margin | present | Present | Absent | Present | Present | Present | Absent | Absent | Present | Pancreatitis from hypercalcaemia secondary to malignancy |
| 34 | iCCA | iCCA | CT | PV only | Present | 14 | Heterogenous mass with irregular margin | Present | Present | Present | Absent | Present | Present | Absent | Absent | Absent | Bone and nodes |
| 35 | Possible iCCA | Possible iCCA | CT and MRI | PV only | Absent | n/a | Heterogenous mass with irregular margin | Present | Present | Present | Absent | Present | Present | Absent | Present | Present | Nodes, peritoneal nodules and small volume ascities |
| 41 | Possible iCCA | iCCA | CT | Unenhanced | Present | 8 | Heterogenous mass with irregular margin | Present | n/a | Absent | Absent | Absent | Absent | Absent | Absent | Absent | Portal nodes |
| 45 | iCCA | iCCA | CT | art, PV | Present | 9 | Heterogenous mass with irregular margin | Present | Present | Absent | Absent | Present | Present | Absent | Absent | Absent | Nodes and lung |
| 48 | iCCA | iCCA | CT and MRI | pv only | Present | 8 | Heterogenous mass with irregular margin | Present | Present | Absent | Present | Present | Present | Absent | Absent | Absent | Bone, nodes, adrenal and small volume ascities |
| 56 | iCCA | iCCA | CT | art, PV | Present | 8 | Heterogenous mass with irregular margin | Present | Present | Present | Present | Present | Present | Absent | Absent | Absent | Nodes |
| 59 | iCCA | iCCA | CT | pv only | Present | 8 | Heterogenous mass with irregular margin | Present | Present | Absent | Present | Present | Present | Present | Absent | Absent | Peritoneal nodules and small volume ascities |
| 60 | iCCA | iCCA | CT | Art, PV | Present | 5 | Heterogenous mass with irregular margin | Present | Present | Present | Present | Present | Absent | Absent | Absent | Absent | Nodes, adrenal and peritoneal nodules |
| 62 | iCCA | iCCA | CT | art, PV | Present | 5 | Heterogenous mass with irregular margin | Present | Present | Absent | Present | Absent | Present | Absent | Absent | Absent | Nodes |
| 67 | iCCA | iCCA | CT | pv only | Present | 9 | Heterogenous mass with irregular margin | Present | Present | Present | Absent | Present | Present | Absent | Present, early | Absent | Bone, nodes and adrenal |
| 70 | iCCA | iCCA | CT and MRI | pv only | Present | 9 | Heterogenous mass with irregular margin | Present on T1 | Present | Present | Absent | Present | Present | Absent | Absent | Absent | Nodal and adrenal |

*iCCA=intrahepatic cholangiocarcinoma; CT=computerised tomography; MRI=magnetic resonant imaging; Art=arterial phase; PV=portal venous phase*

**Supplementary figure 1.**

**A**

(1) Where an accurate date of diagnosis was not available, in line with good practice, the date of biopsy or 15th of the month of diagnosis were used

(2) Performance Status was collected according to the Eastern Cooperative Oncology Group (ECOG) definition, based on the patient's status at presentation, not subsequent statuses collected during treatment

(3) Where the patient or physician concluded that chemotherapy was not appropriate or suitable, the patient was receiving Best Supportive Care

(4) Where patients were receiving radical radiotherapy, this was considered a line of treatment and not inclusive of Best Supportive Care. Where patients were receiving Palliative Radiotherapy, this was included in Best Supportive Care strategy

(5) To calculate survival, where the patient was not deceased, the date of last documented visit as of data lock was used

**B**

| **Classification** | Definition |
|---|---|
| *Non-Evaluable* | No scan available for patient at time of review |
| *iCCA** | "Hypodense hepatic mass in the unenhanced phase with irregular margins, peripheral rim enhancement in the arterial phase and progressive attenuation on venous and delayed phases. CT can also detect the level of biliary obstruction, capsular retraction or hepatic atrophy" (Bridgewater et al. 2014) |
| *Possible iCCA* | Review equivocal- unable to classify as strictly iCCA or no iCCA |
| *Non-iCCA* | Multiple small lesions within the lobes of the liver, in keeping with a metastatic process |

**Supplementary Figure 2**

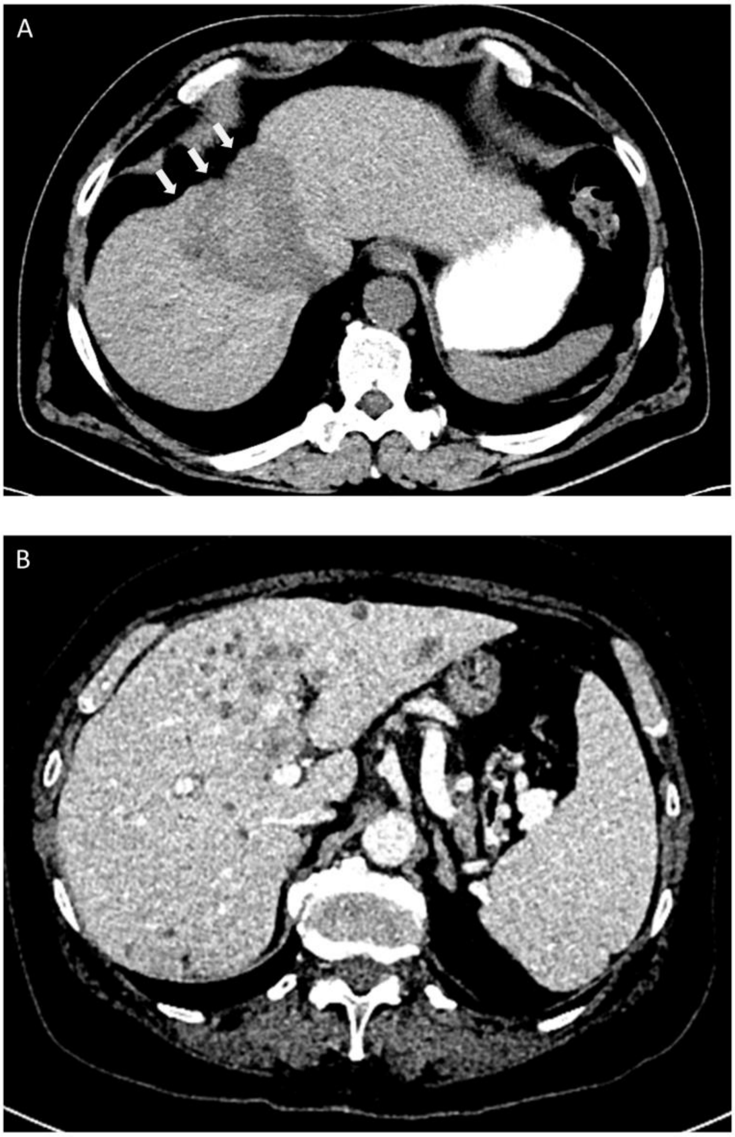

**Supplementary Figure 3**

A

| | Median OS (mths) |
|---|---|
| Primary diagnosis | 18.5 |
| cCUP | 8.8 |
| iCCA | 5.6 |

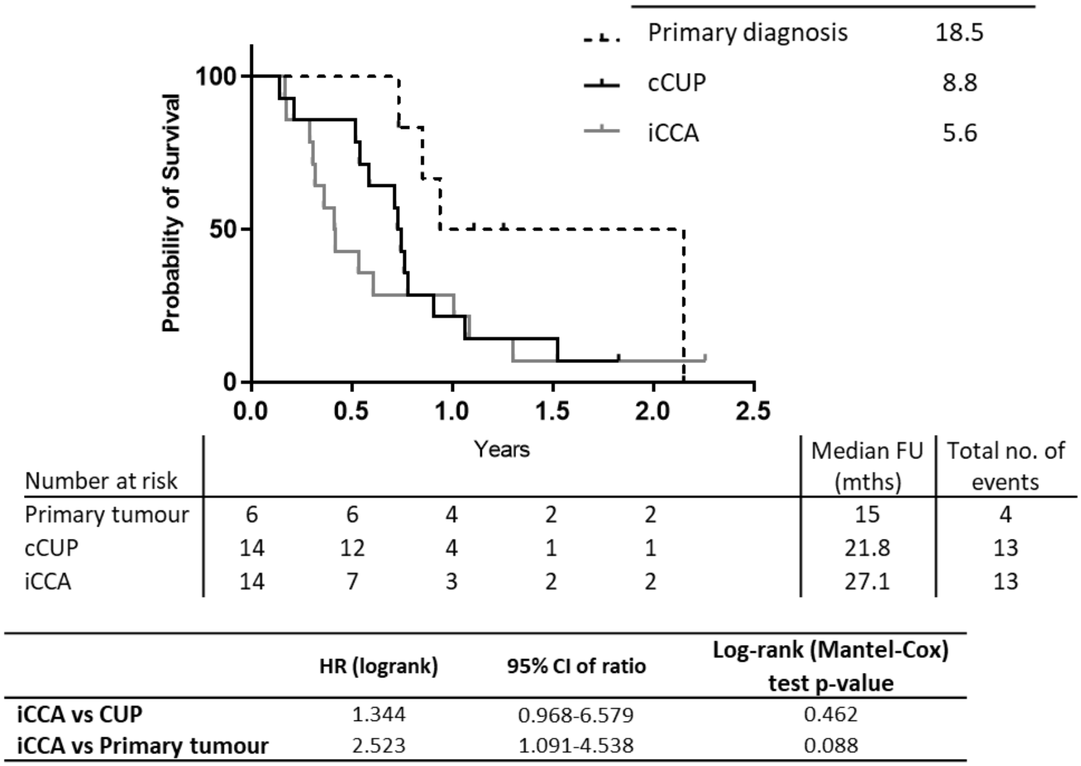


| Number at risk | | | | | | Median FU (mths) | Total no. of events |
|---|---|---|---|---|---|---|---|
| Primary tumour | 6 | 6 | 4 | 2 | 2 | 15 | 4 |
| cCUP | 14 | 12 | 4 | 1 | 1 | 21.8 | 13 |
| iCCA | 14 | 7 | 3 | 2 | 2 | 27.1 | 13 |

| | **HR (logrank)** | **95% CI of ratio** | **Log-rank (Mantel-Cox) test p-value** |
|---|---|---|---|
| **iCCA vs CUP** | 1.344 | 0.968-6.579 | 0.462 |
| **iCCA vs Primary tumour** | 2.523 | 1.091-4.538 | 0.088 |

B

| | Median OS (mths) | HR(log-rank) (95% CI of ratio) | Log-rank (Mantel-Cox) test |
|---|---|---|---|
| cCUP | 8.9 | 0.840 (0.294-2.405) | 0.7387 |
| iCCA | 5.1 | 1.190 (0.412-3.407) | |

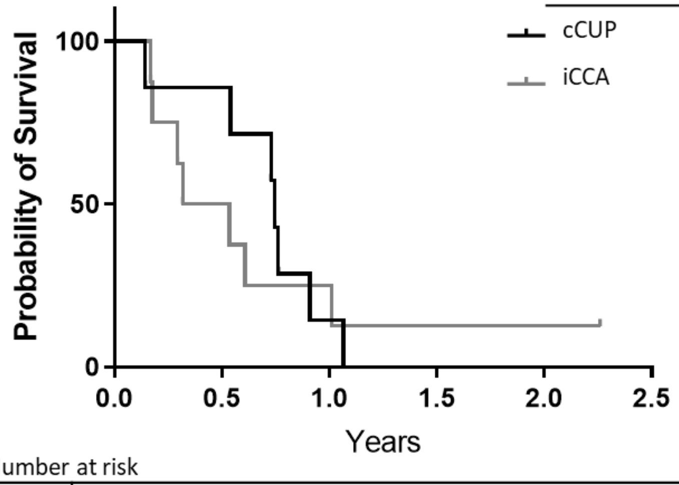


| Number at risk | | | | | | Median FU (mths) | Total no. of events |
|---|---|---|---|---|---|---|---|
| cCUP | 7 | 7 | 2 | 1 | 1 | 8.9 | 7 |
| iCCA | 8 | 4 | 2 | 2 | 1 | 6.4 | 7 |
